# Book Reviews

**Published:** 1911-04

**Authors:** 


### BOOK REVIEWS

COLLECTED PAPERS BY THE STAFF OF ST. MARY'S HOSPITAL, MAYO CLINIC, ROCHESTER, MINNESOTA, 1905-1909. Octavo of 668 pages, illustrated. Philadelphia and London: W. B. Saunders Company, 1911. Cloth $5.50 net.

This interesting book presents in convenient form all of the papers written by the Mayos and their corps of scientific associates and affords an exceedingly valuable work of reference to the many surgical procedures for which these men are so extensively known.

A TREATISE ON DISEASES OF THE NOSE, THROAT AND EAR. By William Lincoln Ballenger, M. D., Professor of Laryngology, Rhinology and Otology in the College of Physicians and Surgeons, Chicago. New (3d) edition, thoroughly revised. Octavo. 983 pages, with 506 engravings, mostly original, and 22 plates. Cloth, $5.50 net. Lea & Febiger, Philadelphia and New York, 1911.

This work has already attained a remarkable record of popularity. Three editions have been called for in less than three years, and during part of this time it has been entirely out of print, owing to the unexpected rapidity of exhaustion. Sustained success is built on merit. Professor Ballenger started his book with adequate preparation. Regardless of labor, he added to his library some 3,000 monographs, pamphlets and reprints, by corresponding with colleagues in America and Europe. In this way he obtained the very latest literature, embracing the most advanced knowledge throughout the civilized world. This he has presented in a most readable form.

---

DISLOCATIONS AND JOINT FRACTURES. By Frederic Jay Cotton, A.M., M.D., First Assistant Surgeon, Boston City Hospital. Octavo of 654 pages, 1201 original illustrations. Philadelphia and London: W. B. Saunders Company, 1910. Cloth. $6.00, net; Half Morocco, $7.50, net.

This splendid monograph cannot be too highly praised. Cotton has presented in a logical and systematic manner vastly more than one somewhat unfamiliar with the subject could imagine was known of this subject. A pleasing and refreshing part is that he has done the work connected with the text as well as illustrations himself rather than by assistants as is so often the case.

---

DIFFERENTIAL DIAGNOSIS. PRESENTED THROUGH AN ANALYSIS OF 383 CASES. By Richard C. Cabot, M.D., Assistant Professor of Clinical Medicine, Harvard Medical School. Octavo of 753 pages, illustrated. Philadelphia and London: W. B. Saunders Company, 1911. Cloth, $5.50, net.

Cabot has departed far from the conventional manner of writing medical books, but has given us a work which, if carefully read should help everyone, physician or surgeon, in that important but difficult part of his work, differential diagnosis. His plan has been to present a list of the common causes of symptoms; these he classified in the order of their frequency and illustrates them by case-histories in which the presenting symptom is followed home until a diagnostic problem and its solution are presented.

---

A TEXT-BOOK OF GENERAL BACTERIOLOGY. By Edwin O. Jordan, Ph.D., Professor of Bacteriology, in the University of Chicago and in Rush Medical College. Second Revised Edition. Octavo of 594 pages, illustrated. Philadelphia and London: W. B. Saunders Company, 1910. Cloth, $3:00, net.

This book is the outgrowth of the lectures given to the students in the University of Chicago during the past few years and affords the student and practitioner an excellent condensed description of this subject.

